# Effects of Different Kinesio-Taping Applications for Delayed Onset Muscle Soreness after High-Intensity Interval Training Exercise: A Randomized Controlled Trial

**DOI:** 10.1155/2021/6676967

**Published:** 2021-06-21

**Authors:** Bao-Lien Hung, Chen-Yu Sun, Nai-Jen Chang, Wen-Dien Chang

**Affiliations:** ^1^Department of Sports Medicine, China Medical University, Taichung, Taiwan; ^2^Department of Sport Performance, National Taiwan University of Sport, Taichung, Taiwan; ^3^Department of Sports Medicine, Kaohsiung Medical University, Kaohsiung, Taiwan

## Abstract

**Objectives:**

Kinesio-taping (KT) is used commonly for the management and prevention of sports injuries. High-intensity interval training (HIIT) is a common muscle strength training and often accompanies delayed onset muscle soreness (DOMS) to interfere with individuals' exercise adherence. So, we compared the effects on muscle pain, thigh edema, and muscle strength for two kinds of KT applications on quadriceps muscles with DOMS after HIIT exercise.

**Methods:**

This is a randomized controlled trial study, which was conducted in a sports medicine laboratory of the college, and all data were collected between February 2019 and February 2020. Healthy participants were recruited from a local university and nearby community by announcements. They were randomly assigned to Group Y (Y-shaped KT application), Group CC (crisscross weave KT application), or Group CON (non-KT). All of them were assessed and used KT following the HIIT exercise, which was used to induce DOMS in the quadriceps muscles. Two different KT applications were, respectively, used in Groups Y and CC, whereas Group CON received no KT application. The visual analog scale (VAS), pressure pain threshold (PPT), thigh circumference, and muscle strength were assessed on the quadriceps femoris muscles before, immediately after, and at 24, 48, and 72 h after exercise.

**Results:**

A total of 38 participants completed the study trial. There were no significant differences in gender, age, height, weight, BMI, body fat, and muscle mass among the three groups (*p* > 0.05). HIIT had a significant impact on muscle soreness, as revealed by the increase in VAS at 24 h after exercise. The results revealed no effect on VAS, PPT, and thigh circumference in Group Y and Group CC (all *p* > 0.05). Additionally, muscle strength was significantly higher in Group CC at 24 h and 48 h after exercise compared with Groups Y and Group CON (*p* < 0.05).

**Conclusion:**

In summary, this experiment reveals no evidence of the effectiveness of Y-shaped and crisscross weave KT applications in the improvement of DOMS pain and edema in the quadriceps muscle. However, the crisscross weave KT application on the quadriceps muscle improved muscle strength recovery after HIIT, but the Y-shaped KT application did not exert this effect. This finding may be useful for muscle strength recovery during HIIT or continuous running competitions.

## 1. Introduction

Delayed onset muscle soreness (DOMS) is characterized and defined by muscle soreness and discomfort, which occurs within 24 h and typically peaks 24 to 48 h after strenuous exercise [1]. High-intensity interval training (HIIT) is the common strenuous exercise to induce DOMS because the exercise process was paired with muscle damage response, resulting in the decrease of muscle strength and increase of muscle soreness [2]. HIIT is performed as short periods of intense exercise interspersed with short-time recovery periods until too exhausted [2]. Athletes, who perform overloaded training or unaccustomed HIIT exercise, also often induce DOMS. Exercise-induced muscle damage often results in DOMS, which is associated with an inflammatory response [3]. The muscle soreness and discomfort of DOMS were relieved over time after strenuous HIIT exercise. Wiewelhove et al. indicted that HITT could induce muscle fatigued and sarcomere length redistribution, resulting in the decrease of metabolism and increase of muscle soreness of DOMS [2]. The discomfort symptoms of DOMS often affect the athletic performance to schedule training plans of athletes [1]. Effective interventions to alleviate the symptoms of DOMS may have positive benefits for the sports population. Various intervention strategies, such as whole-body vibration, ice, and massage, have been used to reduce the symptoms of DOMS [4, 5]. The recovery methods for DOMS are to increase blood flow, improve proprioception to reduce muscle soreness, and eliminate edema associated with fatigued muscle [6, 7]. Numerous interventions, that is, massage, cryotherapy, and stretching, have been used to alleviate the symptoms of DOMS, but small effect size or no evidence has been used to support the positive use of these methods for DOMS [8].

Kinesio-taping (KT) is a conservative treatment for the rehabilitation of musculoskeletal injuries. The KT application is one of the interventions to manage and prevent sports injuries for athletes [9]. Furthermore, it can be used for 3–5 days and dries quickly after contact with water [9]. When KT is applied to the skin along with the injured muscle, the tape elasticity lifts the skin to produce folds, which increased the microcirculation of blood and lymph fluid [10]. So, the effects on stabilizing muscle and reducing pain are popular with athletes. A crisscross weave KT application on the quadriceps muscle is a common method to use for DOMS. This application provided a large lifting skin area under the tape, which increases lymphatic fluid circulation, thereby reducing edema [11, 12]. Pop et al. proved that KT application had a significant effect on edema reduction for mastectomy, and indicated KT is an intervention equally effective as lymphatic drainage [13]. According to KT manufacturers, the KT inhibition technique, in which the tape is applied from muscle insertion to origin, can activate the Golgi tendon organ, thereby inhibiting the overused and stretched muscle [11]. In accordance with the KT inhibition technique, the Y-shaped KT application is another method and frequently used on the quadriceps muscle for treating DOMS in our clinical practice. Physiotherapists and athletic trainers thought that these KT applications may be effective interventions for enhancing muscle recovery and decreasing muscle soreness after exercise. Therefore, the shape and manufacturers were important factors of KT to use for muscular conditions and affect the effects on fatigued muscle recovery.

The cutting shapes of tape according to the KT manufacturers were varied for various muscular conditions. Some study findings have supported, respectively, that Y-shaped or crisscross weave KT application had effects on muscle recovery for fatigued quadriceps muscles after exercise [14, 15]. KT has been proposed positively to use on muscle recovery with different clinical reasoning, and they are therefore plausible to hypothesize that Y-shaped or crisscross weave KT application could decrease muscle soreness and improve symptoms of DOMS. However, no studies have compared the effects of both KT applications on the treatment of DOMS after exercise. So, our study aimed to investigate the effects of KT on quadriceps muscle with DOMS and to clarify whether the effects vary in relation to the different KT applications. We hypothesized that the DOMS-induced muscle pain, thigh edema, and muscle strength in the quadriceps muscles before, immediately after, and after 24, 48, and 72 h after HIIT exercise would be similar in different KT applications.

## 2. Methods

### 2.1. Study Design, Setting, and Sample Size

**Figure 1:**
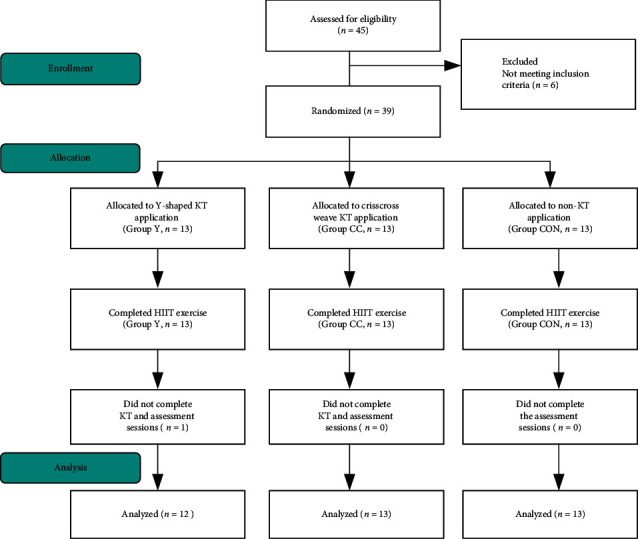
Flowchart diagram of this study.

This is a randomized controlled trial design, which was conducted in a sports medicine laboratory at China Medical University. All data were collected between February 2019 and February 2020. Participants were divided into 3 groups, where Groups Y and CC received Y-shaped and crisscross weave KT, respectively, and Group CON received non-KT as controls (Figure 1). All outcomes were measured before, immediately after, and after 24, 48, and 72 h of DOMS induction by one researcher. Participants were instructed to remove the KT tape, and the visual analog scale (VAS), pressure pain threshold (PPT), thigh circumference, and muscle strength on both the quadriceps femoris muscle were assessed. Immediately after 24, 48, and 72 h of DOMS induction, the KT strip was removed before starting the assessment. After all of the assessments were measured, the KT strip was renewed, following a previous KT application on the quadriceps muscle. The assessment process started with the left leg and then ended with the right leg. The average of measure outcomes from both legs was calculated for analysis. This was an unblinded trial because the participants understood the different KT use, and the assessor knew the participants received which one KT application.

Based on a study conducted by Szymura et al. [16], the G*∗*Power software (version 3.1.9.2; Heinrich-Heine-Universität, Düsseldorf, Germany) used the F test for repeated measures ANOVA and calculated the estimated sample size with *α* level of 0.05 and statistical power of 80%. The estimated sample size was determined at least 13 in each group.

### 2.2. Participant

Healthy participants were recruited from a local university and nearby community by announcements. They were volunteered to participate before the study trial. The inclusion criteria for this study were as follows: healthy individuals and no history of cardiopulmonary, or musculoskeletal disorders. Exclusion criteria were as follows: quadriceps muscle soreness or tenderness; being treated for musculoskeletal conditions at the time of the study; and having previously used KT for DOMS. These medical histories and conditions were inquired to comply with study criteria by a physiotherapist. Participants were randomly assigned to three groups by using a computer-generated random method (1 : 1 : 1 ratio), listing on the randomization number sheet to allocate each participant to the selected group.

### 2.3. Intervention

HIIT exercise was focused on quadriceps muscle training. All of the participants performed 10 min of warm-up exercises, including 3 sets of 30 s of active stretching of the bilateral knee extensors and 5 min walking, before exercise. Then, they performed 5–6 sets of 20 split lunges, 5–6 sets of 20 pulsing squats, and 5–6 sets of 20 squat jumps, 10-sec rest between series, and ended at the point of muscular exhaustion, which was unable to do the exercise. There was an interval of 3 min rest between the two sets of exercises. This protocol of HIIT exercise has been demonstrated to induce DOMS on the quadriceps muscle [17].

**Figure 2:**
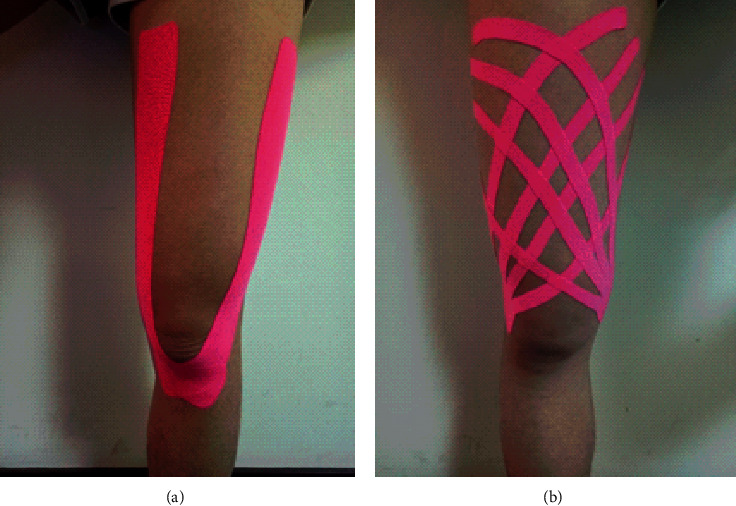
KT applications with Y-shaped (a) and crisscross weave (b).

After the exercise protocol for DOMS induction, an alcohol cotton piece was used to clean the anterior thigh skin of participants, and KT applications on the quadriceps muscle in Groups Y and CC are represented in Figure 2. KT was not applied to participants in the control group. A Kinesio tape with a width of 5 cm (Kinesio Tex Gold, Kinesio, Albuquerque, NM, USA) was used, and the KT application was performed following KT manual guidelines recommended by Kase [11]. Y-shaped and crisscross weave KT were applied to the quadriceps muscles in the supine position in Groups Y and CC, respectively. The length of KT tape was cut as half-length of the participant's thigh (thigh length was measured from the anterior superior iliac crest to tibial tuberosity). Y-shaped KT was used in combination with two original tapes, and crisscross weave KT was used in combination with two 4-tail strips. In Y-shaped KT, the KT tape was strapped to the tibia tuberosity, and the end of the tape was split and strapped on the medial or lateral thigh. In crisscross weave KT, the KT tape was strapped to the medial or lateral femoral condyle and then applied to obliquely cross the thigh. The 4-tail tape end was split and strapped on the contralateral side of though. Based on KT manual guideline [11], both KT methods involved application on the skin, with 5% to 10% tension as clinical experience, from the distal to the proximal thigh. All KT applications were performed by the same researcher. KT was removed after 24 h, renewing the strip during the experiment. Participants were instructed to continue with normal daily activities and stop the use of medicine or other interventions.

### 2.4. Comparison

Before the experimental procedure, the basic data of participants, namely, gender, age, height, weight, BMI, body fat, muscle mass, and degree of exercise participation, were recorded. Body composition, including weight, BMI, body fat, muscle mass, was assessed using a bioelectrical impedance device (TANITA-BC545 body-fat analyser, Tanita Corp., Tokyo, Japan). The degree of exercise participation was used to determine the general physical fitness for guiding HITT exercises, by using the equation proposed by Fox et al.: exercise participation degree = frequency of exercise × (intensity of exercise + duration of exercise) [18]. The range of total exercise participation degree was 2∼72 scores. Exercise frequency is determined by exercise days per week, and scores 1∼6 are represented, respectively, as 0, 1, 2, 3, 4, and >5 days/week. Exercise intensity is confirmed by self-reported fatigue after exercise, and scores 1∼6 are represented as extremely easy, very easy, easy, tired, very tired, and extremely tired, respectively. Exercise duration was assessed by average exercise time per day, and scores 1∼6 are represented, respectively, as 0∼10, 11∼20, 21∼30, 31∼40, 41∼50, and >51 min/day [18]. An exercise protocol was then used to induce DOMS on the bilateral quadriceps muscle. KT was applied on the bilateral fatigued quadriceps muscle for 72 h. The effect on fatigued muscle recovery was determined via VAS, PPT, thigh circumference, and muscle strength before and immediately after 24, 48, and 72 h after HIIT exercise.

#### 2.4.1. Visual Analog Scale

The VAS was used to assess DOMS pain before and after exercise [19]. The VAS is a subjective measurement with verbal descriptors, which is scored from 0 (no pain) to 10 (extremely sore). It for assessing pain intensity had high reliability, which was 0.97 of intraclass correlation coefficients [20]. Participants were asked to rate their muscle soreness during active knee extension without weight loading. They sat and performed to extend their knee from 90° knee flexion to full knee extension 5 times, and the VAS for muscle soreness was measured.

#### 2.4.2. Pressure Pain Threshold

A pain threshold meter (model PTHAF2, American Pain Diagnosis and Thermal Imaging Corporation, Great Neck, NY, USA) was used to assess the PPT of the femoral quadriceps. The PPT is a valid assessment to determine muscle tenderness, and the intraclass correlation coefficient was 0.77, which had good test-retest reliability [21]. It was measured with the participant in a sitting posture. The assessed points were 5, 10, and 15 cm above the apex of the patella along the midline of the thigh. A pressure force was applied to each point at a rate of approximately 1 kg/s [22]. Participants were instructed to report when the feeling first changed from pressure sensation to muscle soreness, and the PPT measure was repeated three times at each point, following the order of 5, 10, and 15 cm above the apex of the patella. The average value of the three times was calculated for each assessed point.

#### 2.4.3. Thigh Circumference

Thigh circumference was used to be a measure of acute change in thigh volume, and the increase of measurement was represented as edema occurrence caused by exercise-induced muscle damage [23]. The reliability of the measurement for thigh muscle volume was high (intraclass correlation coefficient = 1) [24]. It was measured at 5, 10, and 15 cm above the apex of the patella along the midline of the thigh, and the measure order is the same as the PPT measure. The participant was in the supine position, and a roller tape measure (F10-02, Muratec-KDS, Kyoto, Japan) was used to assess each site three times. The average value was calculated for each assessed site.

#### 2.4.4. Muscle Strength

Muscle strength was assessed using the muscular dynamometer (microFET3 Muscle Testing Dynamometer and Inclinometer‚ Hoggan Scientific, LLC. ‚UT' USA), which performs the maximum isometric contraction and assesses the muscle strength recovery [25]. It had excellent *t*-test-retest reliability (intraclass correlation coefficients = 0.70) for muscle force testing [26]. The participant's knee was flexed to a 90-degree position in a sitting position, the dynamometer was placed at the anterior tibia above the ankle joint, and the 5 s maximum isometric contraction for muscle strength measure (Ib) was repeated two times with a 30 s rest between measures. The average value of muscle strength was calculated for analysis.

### 2.5. Ethics and Endpoint

The study procedure was approved by the Institutional Review Board of China Medical University and Hospital (no. CMUH107-REC3-155) and followed the Declaration of Helsinki protocols. All participants provided informed consent before participation in this study. In the data analysis, we used a VAS to measure DOMS after HITT as the primary endpoint, and the effects of PPT, thigh circumference, and muscle strength were examined as the secondary outcomes.

### 2.6. Statistical Analysis

SPSS version 23.0 (IBM Corp., Armonk, NY, USA) was used for statistical analyses. All data are represented using descriptive statistics. The Shapiro–Wilk test was performed to confirm data normality. An independent *t*-test and chi-square test were used to compare participants' characteristics between the groups. The VAS, PPT, thigh circumference, and muscle strength were analyzed using a repeated-measures ANOVA (group × time). Bonferroni post hoc test was used to compare differences in all assessments between the assessed times in each group. Changes between the assessed times are also presented as the mean ± standard error. The significance level was set at *p* < 0.05.

## 3. Results

In this study, we included 39 participants who were divided randomly into three groups. One participant dropped out of Group Y for personal reasons, and the remaining 38 participants completed the study trial without any adverse events. All of the participants gave a positive response to the use of KT tape, and no adverse reaction was reported. As shown in Table 1, no significant differences in gender, age, height, weight, BMI, body fat, and muscle mass were observed between the groups (*p* > 0.05). The total score of exercise participation degree also did not differ significantly between the groups (*p* > 0.05). The thigh-length measure was used to determine the KT tape length.

### 3.1. Visual Analog Scale

**Figure 3:**
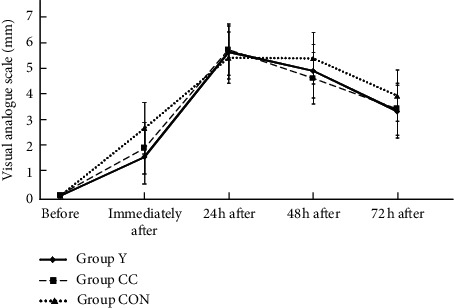
The VAS of 3 groups among before, immediately after, and at 24 hr, 48 hr, and 72 hr after exercise.

The maximum muscle pain of DOMS occurred at 24 h and decreased after 48 and 72 h in all three groups (Figure 3). The main effects of group (*F*_2,35_ = 0.73, *p*=0.48), time (*F*_2,35_ = 290.80, *p*=0.001), and group × time interaction (*F*_2,35_ = 2.18, *p*=0.08) were found. Compared with post-DOMS, VAS score at 24 h after exercise was significantly the highest in all the three groups (Group *Y* = 5.59 ± 1.62; Group CC = 5.70 ± 1.17; and Group CON = 5.37 ± 1.64). In Group CC, the VAS score of 5.70 ± 1.17 at 24 h after exercise was significantly reduced to 4.57 ± 1.91 at 48 h after exercise. VAS measure for muscle soreness was decreased in all the three groups at 72 h after exercise (Group *Y* = 3.28 ± 2.42; Group CC = 3.37 ± 2.18; and Group CON = 3.89 ± 1.55). The VAS values before and immediately after 24, 48, and 72 h after exercise did not differ significantly among the three groups (*p* > 0.05).

### 3.2. Pressure Pain Threshold

**Figure 4:**
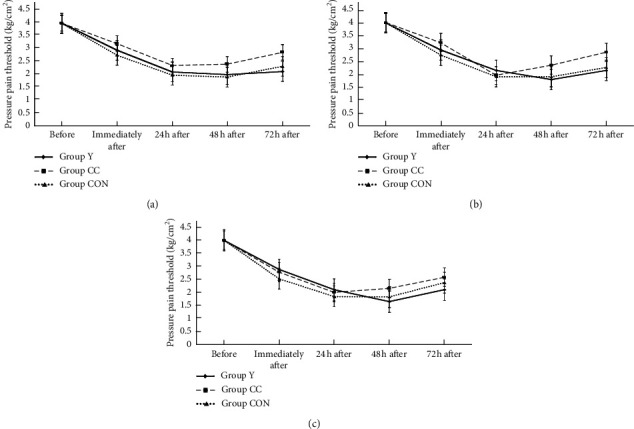
The PPT above 5 cm (a), 10 cm (b), and 15 cm (c) of patella in 3 groups among before, immediately after, and at 24 hr, 48 hr, and 72 hr after exercise.

Figure 4 illustrates that the maximal decrease in PPT was observed 24 and 48 h after exercise. For PPT above 5 cm of the patella, we noted significant main effects of group (*F*_2,35_ = 0.91, *p*=0.41), time (*F*_2,35_ = 131.62, *p*=0.001), and group × time interaction (*F*_2,35_ = 3.22, *p*=0.06). For PPT above 10 cm of the patella, the main effects of group (*F*_2,35_ = 0.86, *p*=0.42), time (*F*_2,35_ = 115.34, *p*=0.001), and group × time interaction (*F*_2,35_ = 1.94, *p*=0.06) were found. For PPT above 15 cm of the patella, the main effects of group (*F*_2,35_ = 0.37, *p*=0.69), time (*F*_2,35_ = 120.63, *p*=0.01), and group × time interaction (*F*_2,35_ = 1.74, *p*=0.09) were represented. The values of PPT above 5, 10, and 15 cm of the patella recovered at 72 h after exercise in three groups. However, PPT values before and immediately after 24, 48, and 72 h after exercise did not differ significantly among the three groups (*p* > 0.05).

### 3.3. Thigh Circumference

For thigh circumference above 5 cm of the patella (Table 2), we noted significant main effects of group (*F*_2,35_ = 1.52, *p*=0.23), time (*F*_2,35_ = 6.12, *p*=0.002), and group × time interaction (*F*_2,35_ = 0.45, *p*=0.87). For thigh circumference above 10 cm of the patella, the main effects of group (*F*_2,35_ = 1.14, *p*=0.32), time (*F*_2,35_ = 7.34, *p*=0.001), and group × time interaction (*F*_2,35_ = 0.42, *p*=0.91) were found. For thigh circumference above 15 cm of the patella, the main effects of group (*F*_2,35_ = 1.25, *p*=0.30), time (*F*_2,35_ = 7.24, *p*=0.001), and group × time interaction (*F*_2,35_ = 0.48, *p*=0.85) were represented. No significant differences among the three groups were identified before and immediately after 24, 48, and 72 h after exercise (*p* > 0.05).

### 3.4. Muscle Strength

**Figure 5:**
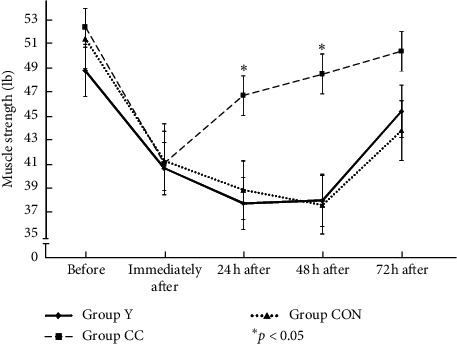
The muscle strength of 3 groups among before, immediately after, and at 24 hr, 48 hr, and 72 hr after exercise. ^*∗*^*p* < 0.05, Group CC versus Group Y or Group CON.

For muscle strength, we found significant main effects of group (*F*_2,35_ = 1.48, *p*=0.24), time (*F*_2,35_ = 37.23, *p*=0.001), and group × time interaction (*F*_2,35_ = 6.04, *p*=0.001). In Figure 5, a reduction in muscle strength was observed immediately after exercise in all three groups. Higher recovery of muscle strength 24 h after exercise was observed 46.65 ± 11.37 Ib in Group CC, which was significantly higher than the other two groups (Group *Y* = 37.70 ± 10.87 Ib; Group CON = 38.80 ± 11.24 Ib) after 24 h after exercise. At 48 h after exercise, the recovery of muscle strength of 48.44 ± 11.98 in Group CC was also significantly higher than the other two groups (Group *Y* = 37.95 ± 15.29 Ib; Group CON = 37.58 ± 11.13 Ib, *p* < 0.05, resp.).

## 4. Discussion

HIIT is a common muscle strength training for athletes and general peoples, which accompanies the muscle soreness of DOMS to interfere the exercise adherence. This is the first study to used crisscross weaved or Y-shaped KT during DOMS muscle recovery after HIIT. We investigated the effects of two cutting shapes of KT according to the user manufacturers on muscle soreness, edema, and strength after DOMS induction. Our results indicated that recovery from muscle soreness occurred at 48 h after DOMS induction, but VAS scores did not have a significant difference between both types of KT applications on the quadriceps femoris muscle. Compared with the control group, no significant differences in crisscross weaved or Y-shaped KT application were also observed in VAS, PPT, and thigh circumference at 24, 48, and 72 h after HIIT exercise.

### 4.1. No Effects of DOMS Muscle Soreness Undergoing Crisscross Weave and Y-Shaped KT

No significant changes in VAS and PPT scores were observed immediately after 24, 48, and 72 h after HIIT between KT and non-KT applications. Some studies have reported that pain in the injured muscle decreases immediately and over a few days following a KT intervention [27, 28]. The functional activity of individuals with muscle pain caused by injured muscles may affect pain reduction by KT intervention [28]. A systematic review further reported statistical evidence of the effects of KT in reducing pain intensity among individuals with myofascial pain syndrome [29]. Their findings supported that, comparing with other interventions, the KT application had significantly positive effects to manage the pain intensity at postintervention and follow-up [29]. However, the analgesic effect of KT intervention on DOMS remained unclear. Lee and Lim reported that muscle pain in the fatigued calf and rectus femoris muscles had not ameliorated 24 h after the KT application [30]. Kirmizigil reported that the KT application was favorable in muscle soreness recovery from DOMS but did not exhibit a significant effect on VAS scores compared with the non-KT group [31]. The VAS is a subjective and self-report measurement for muscle pain and thus has measure variability, due to differences in an individual's perception of pain intensity [19]. The PPT is used to measure muscle pain recovery after DOMS in the current study, and pressure algometry is a type of high validity and reliability tool [32]. Our results found HIIT had a significant impact on DOMS muscle soreness, as revealed by the increase in VAS and decrease in PPT at 24 h after exercise.

Ozmen et al. used Y-shaped KT on the quadriceps femoris muscles with DOMS and did not observe an increased reduction in VAS after 2 days of recovery compared with the non-KT group [33]. This outcome is similar to our results. Ozmen et al. also concluded that KT was not an effective intervention for muscle pain relief [33]. Some studies have demonstrated that KT application affects DOMS in a decrease of muscle tension [27, 34, 35]. Merino-Marban et al. used KT on DOMS in the calf muscles in athletes and reported it had effects on controlling an increase of muscle pain after the strenuous exercise [27]. The analgesic effect of KT has been demonstrated to be caused by the stimulation of the Golgi tendon organ, which increases the autogenic inhibition and metabolic activity of the muscle tissue [34, 35]. But our result did not reveal an analgesic effect of KT on DOMS in the quadriceps femoris. This outcome differs from those reported by Fratocchi et al. and Bae et al., wherein they applied KT to DOMS in the biceps brachii [34, 35]. However, while the participants in our study caused DOMS on quadriceps femoris muscle after HITT exercise, they still need to use lower extremities for daily physical activities or sport. Boobphachart et al. applied Y-shaped KT on the quadriceps femoris with DOMS and determined that muscle soreness significantly decreased 72 h after exercise compared with the control group [36]. KT increased blood and lymphatic flow under the skin, resulting in decreased muscle soreness and ameliorated inflammatory response of fatigued muscle [11, 12]. The covering area of KT on the skin could be elevated and increased the subcutaneous space, which improved blood microcirculation to remove pain substances [37]. But, some studies have reported no significant reduction in DOMS pain in the KT group after applying Y-shaped KT on the quadriceps femoris [31, 33]. However, the present study did not provide evidence on the effectiveness of crisscross weave and Y-shaped KT applications in the relief of DOMS muscle soreness in quadriceps femoris muscle.

### 4.2. No Effects of DOMS Edema Undergoing Crisscross Weave and Y-Shaped KT

In the current study, we compared two types of KT application on the quadriceps femoris with DOMS. The crisscross weave of KT covered a larger skin area than did the Y-shaped KT. Therefore, the crisscross weave of KT has been used in clinical practice to reduce edema and improve blood circulation in the damaged muscle. However, we observed no changes in the reduction of pain and edema between the two types of KT groups immediately after exercise and on 24–72 h after exercise or compared with the control group. In the cross-over study of Kirmizigil et al., the crisscross weave of KT was applied to the quadriceps femoris with DOMS [31]. They also determined that pain from DOMS decreased at 48 and 72 h after exercise with no difference from the non-KT control group. However, the effects of KT on increasing the covered skin area and increasing blood microcirculation did not appear in this study, because increases in thigh circumference did not occur after HITT exercise in three groups.

### 4.3. Positive Effects on Muscle Strength Recovery of DOMS Undergoing Crisscross Weave KT

Previous studies have reported that the KT application did not improve muscle performance in healthy individuals, regardless of the different types of KT used [38, 39]. The use of the KT facilitation technique, applied from the muscle origin to insertion, could improve muscle contraction and muscle strength [11]. Ozmen et al. used Y-shaped KT on the quadriceps femoris, applied from the muscle origin to insertion [33]. No significant difference in sprint performance was observed between the KT and non-KT groups [33]. In the present study, the KT inhibition technique was applied from the muscle insertion to the origin to inhibit overuse and muscle stretching for DOMS [40]. Kirmizigil et al. used the KT inhibition technique on the rectus femoris muscle with DOMS, but no significant differences in sprint and horizontal jump performance were observed to compare the nontaping group after DOMS induction [31]. Vercelli et al. compared the immediate effects of KT facilitation and inhibition techniques and measured the isokinetic concentric maximal strength of the quadriceps muscle, which revealed no significant differences [40]. Our findings are in accordance with the results of these studies [31, 40] because a Y-shaped KT with the inhibition technique on the quadriceps muscle did not affect muscle strength recovery after exercise.

In contrast to findings regarding the Y-shaped KT application, we observed that the crisscross weave KT application improved muscle strength recovery after exercise. The crisscross weave technique of KT was used to reduce edema and covered a larger area of the fatigued muscle [41]. We presumed that crisscross weave KT application lifted the skin to increase the space, increasing blood circulation by stimulating the vasomotor reflex [42]. This application could also increase muscle metabolism and stimulate the cutaneous fusimotor reflex, causing muscle contraction [43]. The crisscross weave KT application might have improved muscle strength recovery in the present study because an increase in the skin space to improve metabolism was presumed. However, a few studies have focused on the crisscross weave KT application on the fatigued muscle, and further research is needed to clarify its effects on DOMS.

Our study also has limitations. First, the assessment and KT applications were not blinded. A blinded clinical trial was needed to minimize study bias and provide a more complete understanding of KT effectiveness. Second, large and multiple-term trials were inadequate, which decrease the statistical power to prove the benefits of KT. Third, there was no placebo or sham group in the current study. An increase of a placebo or sham group could have been favorable to compare the psychological response to KT. Fourth, the sample population in this study included healthy individuals, so our study results may not be generalized to athletes who suffered DOMS-induced symptoms after HITT training.

## 5. Conclusion

To our knowledge, this is the first randomized controlled trial comparing the effects of Y-shaped and crisscross weave KT applications on DOMS. Our findings revealed that both KT applications did not improve VAS, PPT, and thigh circumference at time points up to 72 h after HIIT. The current study did not provide direct evidence on the effectiveness of both KT applications in the improvement of DOMS pain and edema in the quadriceps muscle. However, the crisscross weave KT application on the quadriceps muscle improved muscle strength recovery at 24 and 48 h after HIIT, but the Y-shaped KT application did not exert this effect. The crisscross weave KT application on quadriceps muscle may be used for fatigued muscle strength recovery for athletics especially during HIIT or continuous running competitions. Further research on the effects of different KT applications on DOMS recovery after exercise is needed.

## Figures and Tables

**Table 1:** Demographic data of the participants.

	Group Y (*n* = 12)	Group CC (*n* = 13)	Group CON (*n* = 13)
Male/female	3/9	6/7	6/7
Age (years)	30.25 ± 11.10	24.54 ± 5.13	29.15 ± 9.91
Height (cm)	163.56 ± 7.78	164.58 ± 6.00	162.81 ± 8.09
Weight (kg)	62.06 ± 9.42	60.49 ± 8.21	60.98 ± 9.82
BMI (kg/m^2^)	23.14 ± 2.63	22.27 ± 2.19	23.00 ± 3.38
Body fat (%)	28.10 ± 5.00	24.25 ± 5.79	28.15 ± 5.27
Muscle mass (kg)	3.73 ± 0.44	4.10 ± 0.54	3.92 ± 0.49
Thigh length (cm)	51.35 ± 3.05	51.51 ± 2.98	50.70 ± 2.98
Exercise participation degree	33.95 ± 41.63	30.33 ± 33.28	37.85 ± 48.56

**Table 2:** The changes on thigh circumference before, immediately after, and at 24 hr, 48 hr, and 72 hr after exercise.

	Group Y (*n* = 12)	Group CC (*n* = 13)	Group CON (*n* = 13)
*Thigh circumference above 5 cm of patella*			
Before exercise (cm)	41.20 ± 2.45	40.41 ± 2.51	41.72 ± 3.51
Immediately after exercise (cm)	41.58 ± 2.57	40.77 ± 2.12	42.00 ± 3.75
24 h after exercise (cm)	41.61 ± 2.60	40.87 ± 2.11	41.97 ± 3.76
48 h after exercise (cm)	41.72 ± 2.47	40.96 ± 2.16	42.13 ± 3.71
72 h after exercise (cm)	41.57 ± 2.32	40.90 ± 2.02	41.99 ± 3.65

*Thigh circumference above 10 cm of patella*			
Before exercise (cm)	45.59 ± 2.49	44.64 ± 2.52	46.26 ± 4.32
Immediately after exercise (cm)	45.97 ± 2.63	44.97 ± 2.41	46.38 ± 4.60
24 h after exercise (cm)	46.00 ± 2.53	45.12 ± 2.48	46.41 ± 4.73
48 h after exercise (cm)	46.10 ± 2.47	45.18 ± 2.54	46.57 ± 4.62
72 h after exercise (cm)	46.04 ± 2.37	45.05 ± 2.35	46.45 ± 4.58

*Thigh circumference above 15 cm of patella*			
Before exercise (cm)	49.68 ± 2.88	48.62 ± 2.86	50.10 ± 4.48
Immediately after exercise (cm)	50.00 ± 2.80	49.00 ± 2.81	50.31 ± 4.90
24 h after exercise (cm)	50.41 ± 2.98	49.12 ± 2.73	50.47 ± 4.94
48 h after exercise (cm)	50.21 ± 2.76	49.26 ± 2.83	50.65 ± 4.96
72 h after exercise (cm)	50.23 ± 2.67	49.11 ± 2.70	50.48 ± 4.91

## Data Availability

The data used to support the findings of this study are included within the article.

## References

[1] Lee Y. S., Bae S. H., Hwang J. A., Kim K. Y. (2015). The effects of kinesio taping on architecture, strength and pain of muscles in delayed onset muscle soreness of biceps brachii. *Journal of Physical Therapy Science*.

[2] Wiewelhove T., Raeder C., Meyer T., Kellmann M., Pfeiffer M., Ferrauti A. (2015). Markers for routine assessment of fatigue and recovery in male and female team sport athletes during high-intensity interval training. *PLoS One*.

[3] Hyldahl R. D., Hubal M. J. (2014). Lengthening our perspective: morphological, cellular, and molecular responses to eccentric exercise. *Muscle & Nerve*.

[4] Veqar Z., Imtiyaz S. (2014). Vibration therapy in management of delayed onset muscle soreness (DOMS). *Journal of Clinical and Diagnostic Research*.

[5] Zainuddin Z., Newton M., Sacco P., Nosaka K. (2005). Effects of massage on delayed-onset muscle soreness, swelling, and recovery of muscle function. *Journal of Athletic Training*.

[6] Connolly D. A. J., Sayers S. E., McHugh M. P. (2003). Treatment and prevention of delayed onset muscle soreness. *Journal of Strength and Conditioning Research*.

[7] Heiss R., Lutter C., Freiwald J. (2019). Advances in delayed-onset muscle soreness (DOMS)-part II: treatment and prevention. *Sportverletzung Sportschaden: Organ der Gesellschaft Fur Orthopadisch-Traumatologische Sportmedizin*.

[8] Torres R., Ribeiro F., Alberto Duarte J., Cabri J. M. H. (2012). Evidence of the physiotherapeutic interventions used currently after exercise-induced muscle damage: systematic review and meta-analysis. *Physical Therapy in Sport*.

[9] Aktas G., Baltaci G. (2011). Does kinesiotaping increase knee muscles strength and functional performance?. *Isokinetics and Exercise Science*.

[10] Kafa N., Citaker S., Omeroglu S., Peker T., Coskun N., Diker S. (2015). Effects of kinesiologic taping on epidermal-dermal distance, pain, edema and inflammation after experimentally induced soft tissue trauma. *Physiotherapy Theory and Practice*.

[11] Kase K., Wallis J., Kase T. (2003). *Clinical Therapeutic Applications of the Kinesio Taping Method*.

[12] Álvarez-Álvarez S., San José F. G.-M., Rodríguez-Fernández A. L., Güeita-Rodríguez J., Waller B. J. (2014). Effects of Kinesio® Tape in low back muscle fatigue: randomized, controlled, doubled-blinded clinical trial on healthy subjects. *Journal of Back and Musculoskeletal Rehabilitation*.

[13] Pop T. B., Karczmarek-Borowska B., Tymczak M., Hałas I., Banaś J. (2014). The influence of Kinesiology Taping on the reduction of lymphoedema among women after mastectomy - preliminary study. *Współczesna Onkologia*.

[14] Choi I.-R., Lee J.-H. (2019). The effect of the application direction of the kinesiology tape on the strength of fatigued quadriceps muscles in athletes. *Research in Sports Medicine*.

[15] Haksever B., Kinikli G. İ., Bayrakçi Tunay V., Karahan S., Dönmez G. (2016). Effect of kinesiotaping intervention on knee muscle strength and delayed onset muscle soreness pain following eccentric fatigue training. *Türk Fizyoterapi Ve Rehabilitasyon Dergisi*.

[16] Szymura J., Maciejczyk M., Wiecek M. (2016). Effects of kinesio taping on anaerobic power recovery after eccentric exercise. *Research in Sports Medicine (Print)*.

[17] Laffaye G., Da Silva D. T., Delafontaine A. (2019). Self-myofascial release effect with foam rolling on recovery after high-intensity interval training. *Frontiers in Physiology*.

[18] Fox K. R. (1999). The influence of physical activity on mental well-being. *Public Health Nutrition*.

[19] Kanda K., Sugama K., Hayashida H. (2013). Eccentric exercise-induced delayed-onset muscle soreness and changes in markers of muscle damage and inflammation. *Exercise Immunology Review*.

[20] Bijur P. E., Silver W., Gallagher E. J. (2001). Reliability of the visual analog scale for measurement of acute pain. *Academic Emergency Medicine*.

[21] Bisset L. M., Evans K., Tuttle N. (2015). Reliability of 2 protocols for assessing pressure pain threshold in healthy young adults. *Journal of Manipulative and Physiological Therapeutics*.

[22] Chesterton L. S., Sim J., Wright C. C., Foster N. E. (2007). Interrater reliability of algometry in measuring pressure pain thresholds in healthy humans, using multiple raters. *The Clinical Journal of Pain*.

[23] Fielding R. A., Violan M. A., Svetkey L. (2000). Effects of prior exercise on eccentric exercise-induced neutrophilia and enzyme release. *Medicine & Science in Sports & Exercise*.

[24] Vaile J. M., Gill N. D., Blazevich A. J. (2007). The effect of contrast water therapy on symptoms of delayed onset muscle soreness. *Journal of Strength and Conditioning Research*.

[25] Muanjai P., Namsawang J. (2015). Effects of stretching and cold-water immersion on functional signs of muscle soreness following plyometric training. *Journal of Physical Education and Sport*.

[26] Harris-Hayes M., Mueller M. J., Sahrmann S. A. (2014). Persons with chronic hip joint pain exhibit reduced hip muscle strength. *Journal of Orthopaedic & Sports Physical Therapy*.

[27] Merino-Marban R., Mayorga-Vega D., Fernandez-Rodriguez E. (2013). Effect of kinesio tape application on calf pain and ankle range of motion in duathletes. *Journal of Human Kinetics*.

[28] Kaya E., Zinnuroglu M., Tugcu I. (2011). Kinesio taping compared to physical therapy modalities for the treatment of shoulder impingement syndrome. *Clinical Rheumatology*.

[29] Zhang X.-F., Liu L., Wang B.-B., Liu X., Li P. (2019). Evidence for kinesio taping in management of myofascial pain syndrome: a systematic review and meta-analysis. *Clinical Rehabilitation*.

[30] Lee H., Lim H. (2020). Effects of double-taped kinesio taping on pain and functional performance due to muscle fatigue in young males: a randomized controlled trial. *International Journal of Environmental Research and Public Health*.

[31] Kirmizigil B., Chauchat J. R., Yalciner O., Iyigun G., Angin E., Baltaci G. (2019). The effectiveness of kinesio taping in recovering from delayed onset muscle soreness: a cross-over study. *Journal of Sport Rehabilitation*.

[32] Kinser A. M., Sands W. A., Stone M. H. (2009). Reliability and validity of a pressure algometer. *Journal of Strength and Conditioning Research*.

[33] Ozmen T., Aydogmus M., Dogan H., Acar D., Zoroglu T., Willems M. (2016). The effect of kinesio taping on muscle pain, sprint performance, and flexibility in recovery from squat exercise in young adult women. *Journal of Sport Rehabilitation*.

[34] Fratocchi G., Di Mattia F., Rossi R., Mangone M., Santilli V., Paoloni M. (2013). Influence of kinesio taping applied over biceps brachii on isokinetic elbow peak torque. A placebo controlled study in a population of young healthy subjects. *Journal of Science and Medicine in Sport*.

[35] Bae S. H., Lee Y. S., Kim G. D., Kim K. Y. (2014). A quantitative evaluation of delayed onset muscular soreness according to application of kinesio taping. *Advanced Science and Technology Letters*.

[36] Boobphachart D., Manimmanakorn N., Manimmanakorn A., Thuwakum W., Hamlin M. J. (2017). Effects of elastic taping, non-elastic taping and static stretching on recovery after intensive eccentric exercise. *Research in Sports Medicine*.

[37] Thelen M. D., Dauber J. A., Stoneman P. D. (2008). The clinical efficacy of kinesio tape for shoulder pain: a randomized, double-blinded, clinical trial. *Journal of Orthopaedic & Sports Physical Therapy*.

[38] Mendez-Rebolledo G., Ramirez-Campillo R., Guzman-Muñoz E., Gatica-Rojas V., Dabanch-Santis A., Diaz-Valenzuela F. (2018). Short-term effects of kinesio taping on muscle recruitment order during a vertical jump: a pilot study. *Journal of Sport Rehabilitation*.

[39] Cheung R. T. H., Yau Q. K. C., Wong K. (2016). Kinesiology tape does not promote vertical jumping performance: a deceptive crossover trial. *Manual Therapy*.

[40] Vercelli S., Sartorio F., Foti C. (2012). Immediate effects of kinesiotaping on quadriceps muscle strength: a single-blind, placebo-controlled crossover trial. *Clinical Journal of Sport Medicine*.

[41] Hazar Kanik Z., Citaker S., Yilmaz Demirtas C., Celik Bukan N., Celik B., Gunaydin G. (2019). Effects of kinesio taping on the relief of delayed onset muscle soreness: a randomized, placebo-controlled trial. *Journal of Sport Rehabilitation*.

[42] Bae S.-H., Lee Y.-S., Kim G.-D., Kim K.-Y. (2014). The effects of kinesio-taping applied to delayed onset muscle soreness on changes in pain. *International Journal of Bio-Science and Bio-Technology*.

[43] Zhang S., Fu W., Pan J., Wang L., Xia R., Liu Y. (2016). Acute effects of Kinesio taping on muscle strength and fatigue in the forearm of tennis players. *Journal of Science and Medicine in Sport*.

